# 915 Developing a Burn Advanced Practice Provider Team: Actualizing the Vision

**DOI:** 10.1093/jbcr/iraf019.446

**Published:** 2025-04-01

**Authors:** Crystal Szczepanski, Yuk Ming Liu, Lauren Nosanov, Laura Johnson

**Affiliations:** Walter L. Ingram Burn Center at Grady Memorial Hospital; Emory University School of Medicine; Walter L. Ingram Burn Center at Grady Memorial Hospital; Walter L. Ingram Burn Center at Grady Memorial Hospital

## Abstract

**Introduction:**

Burn workforce challenges, including an ongoing physician shortage, residency work hour restrictions, and increased hospital admissions have significantly contributed to the need for advanced practice providers (APPs). As in other specialties, the Burn community has recognized the need for integration of APPs into their programs, noting that APPs serve as integral members of the team and contribute to an increase in positive patient outcomes. Our burn center, which averages over 600 admissions a year, serves a large urban community as well as a wide loco-regional area. In the recent 15 years, our Burn team included one, and at times, two APPs assisting in the outpatient clinic and operating room. In anticipation of growth, it was determined that 12 APPs would be required to meet patient care needs in a 24-7 staffing model.

**Methods:**

In coordination with other APP-run critical care units, an onboarding and orientation process was developed. Comprehensive burn and ICU topics are covered during the orientation period through various teaching methods, including participation in wound rounds to provide a more robust wound care experience. The breadth of these topics addresses the learning needs of new graduates and new-to-burns APPs as well as ICU experienced APPs.

**Results:**

Between May 2023 and August 2024, 9 of the 12 positions have been filled with new hires taking advantage of the new orientation process. Core day and night coverage is now able to be provided by APPs with management of patients in the ICU, floor, clinic, OR, and trauma center in collaboration with the monthly rotating resident team.

**Conclusions:**

Well-defined resident and APP service objectives allow for responsibility sharing and education balances between the APPs and resident trainees. The APPs also share in educating medical students, interns, and Critical Care APP Fellow who rotate through the burn center. In addition to patient care, the APPs are encouraged to partake in activities that will guide them in their professional development pathways and allow them to make and achieve future goals. These include program development, education, advocacy and outreach, and peer support. Through developing this program, we hoped to create an environment of retention, job satisfaction, and excellent patient care and to promote involvement in the national burn community.

**Applicability of Research to Practice:**

Successful models of APP-based team development must be studied in order to provide further guidance as practice paradigms shift nationally. Growth of APP involvement in the national burn community would be served by national application of a similar program.

**Funding for the Study:**

N/A

